# Optimal ${\mathscr{P}}{\mathscr{T}}$ -symmetric switch features exceptional point

**DOI:** 10.1038/s41598-017-13264-9

**Published:** 2017-10-16

**Authors:** Anatole Lupu, Vladimir V. Konotop, Henri Benisty

**Affiliations:** 1 Centre de Nanosciences et de Nanotechnologies, CNRS, Univ. Paris-Sud, Université Paris-Saclay, C2N–Orsay, 91405 Orsay, cedex France; 2 Centro de Física Teórica e Computacional and Departamento de Física, Faculdade de Ciências, Universidade de Lisboa, Campo Grande 2, Edifício C8, Lisboa, 1749-016 Portugal; 3 Laboratoire Charles Fabry, Institut d’Optique Graduate School, CNRS, Univ. Paris Saclay, 2 Ave Augustin Fresnel, 91127 Palaiseau Cedex, France

## Abstract

We consider the optimization problem of least energy-cost path in open systems that are described by non-Hermitian Hamiltonians. We apply it to find the optimal gain-loss profile for a non-uniform *PT*-symmetric coupler performing a binary transfer function. We bring evidence that the gain-loss profile fulfilling this requirement corresponds to a non-conventional situation where light intensity is conserved at every point along the *PT*-symmetric system. Besides, we find that the optimal profile corresponds to a practically important case of optical switching operation achieved with minimal amount of aggregate amplification level. We show that switching architectures using such type of gain-loss profiles are much more advantageous than conventional uniform *PT*-symmetric couplers in terms of gain and energy. Furthermore, this type of optimal profile turns out to be robust against fabrication imperfections. This opens new prospects for functional applications of *PT*-symmetric devices in photonics.

## Introduction

## ${\mathscr{P}}{\mathscr{T}}$-symmetry and optimization

The concept of parity-time (${\mathscr{P}}{\mathscr{T}}$) symmetric Hamiltonians, introduced in the seminal paper by Bender and Boettcher^1^, quickly became a new paradigm in the theory of quantum systems^2,3^. It is also currently being extended to a variety of physics branches tackling linear and nonlinear wave physics^4^. Optics became a preferred playground for such extensions. It offered the systems of choice to emulate the equivalent of the complex-valued potential thanks to optical gain and losses of macroscopic photonic systems: specifically, balanced gain and loss can realize ${\mathscr{P}}{\mathscr{T}}$-symmetry, as was first suggested in^5^ and experimentally investigated in the following years^6–8^.

The use of ${\mathscr{P}}{\mathscr{T}}$-symmetric^9^, and even of more general non-Hermitian^10,11^ Hamiltonians, offered new insights into solution of the quantum brachistochrone problem^12^ (fundamentals of the theory remaining a debatable issue^13^). In classical physics the brachistochrone or shortest-time-delay problem is the well-known example of optimization problems^14^. Formally, its quantum version could lead to arbitrarily small evolution times between specific states (in a realistic situation physical constraints impose a lower bound for the transmission time, see e.g^15^.). The vanishing optimal passage time was conjectured in^16^ to be a general feature of non-Hermitian systems related to the existence of an exceptional point, which is the point of the coalescence of eigenvalues and eigenfunctions of the operator^15,17^.

Optimization problems of different kinds arise in non-Hermitian optics, notably because of the importance of energy minimization constraints. Energy costs are a general concern, increasingly so for the management of optical networks. Indeed, the application that we shall consider is an optical switch^18–20^ aimed at routing optical data. It is logical to wonder whether one can minimize the “cost” of operating a ${\mathscr{P}}{\mathscr{T}}$-symmetric switch. In this Letter, we lay the problem as an optimization problem that spans both non-Hermitian and conservative (Hermitian) operators, a new class of problems. Our cost is the total gain needed to achieve switching, i.e. the integral ${\rm{\Gamma }}={\int }_{-{L}_{c}}^{{L}_{c}}\gamma (z)dz$ of the local gain $\gamma (z)\ge 0$, specified below, between the two ends of the device ±*L*_*c*_.

## Model and mathematical optimization of a ${\mathscr{P}}{\mathscr{T}}$-symmetric coupler

We consider two scalar fields *q*_1_ and *q*_2_ propagating in two coupled waveguides, one with gain and the other with balanced losses. The first and second waveguides are respectively subject to gain and loss, which are varying in space and described by a non-negative function $\gamma (z)\ge 0$. Thus we deal with the system:

1 $$i{\dot{q}}_{1}=\kappa {q}_{2}+i\gamma (z){q}_{1},\quad i{\dot{q}}_{2}=\kappa {q}_{1}-i\gamma (z){q}_{2}$$

where an overdot stands for the derivative with respect to *z* and where *κ* is the coupling constant. At constant gain and losses the system (1), intensively studied for more than two decades, see^21^, represents the simplest discrete ${\mathscr{P}}{\mathscr{T}}$-symmetric system, which describes coupled beams in waveguides with a complex Bragg grating^22,23^, two coupled waveguides each one having gain and losses^24^, or two coupled waveguides with gain and loss^6,25^ or with unbalanced losses as in the first experiments on ${\mathscr{P}}{\mathscr{T}}$-symmetry in optics^7,8^ (see also^4^). The model (1) was also considered with gain and losses varying along the propagation distance: pulse switching on localized gain-and-loss elements^26^, level crossing in a two-level system subject to periodically varying gain-and-loss^27^, parametric oscillations in locally ${\mathscr{P}}{\mathscr{T}}$-symmetric systems^28^, statistics of the field distribution in a coupler with randomly varying gain and losses^29^.

Before going into details of the analysis, we notice that the optimization results presented below are also valid for a more general system with still $i{\dot{q}}_{1}=\kappa {q}_{2}+i{\gamma }_{1}(z){q}_{1}$, $i{\dot{q}}_{2}=\kappa {q}_{1}-i{\gamma }_{2}(z){q}_{2}$ where however gain ${\gamma }_{1}(z)\ge 0$ and losses ${\gamma }_{2}(z)\ge 0$ are not equal, i.e. ${\gamma }_{1}(z)\ne \gamma (z)$ or ${\gamma }_{2}(z)\ne \gamma (z)$. Indeed, denoting $[{\gamma }_{1}(z)-{\gamma }_{2}(z)]/2=g(z)$, and introducing ${\mathop{q}\limits^{ \sim }}_{j}={q}_{j}\exp (\frac{1}{2}{\int }_{-{L}_{c}}^{z}[{\gamma }_{2}(\zeta )-{\gamma }_{1}(\zeta )]d\zeta )$, one can verify that ${\tilde{q}}_{\mathrm{1,2}}$ solve the system (1) with $\gamma (z)=$ $[{\gamma }_{1}(z)+{\gamma }_{2}(z)]/2$.

**Figure 1:**
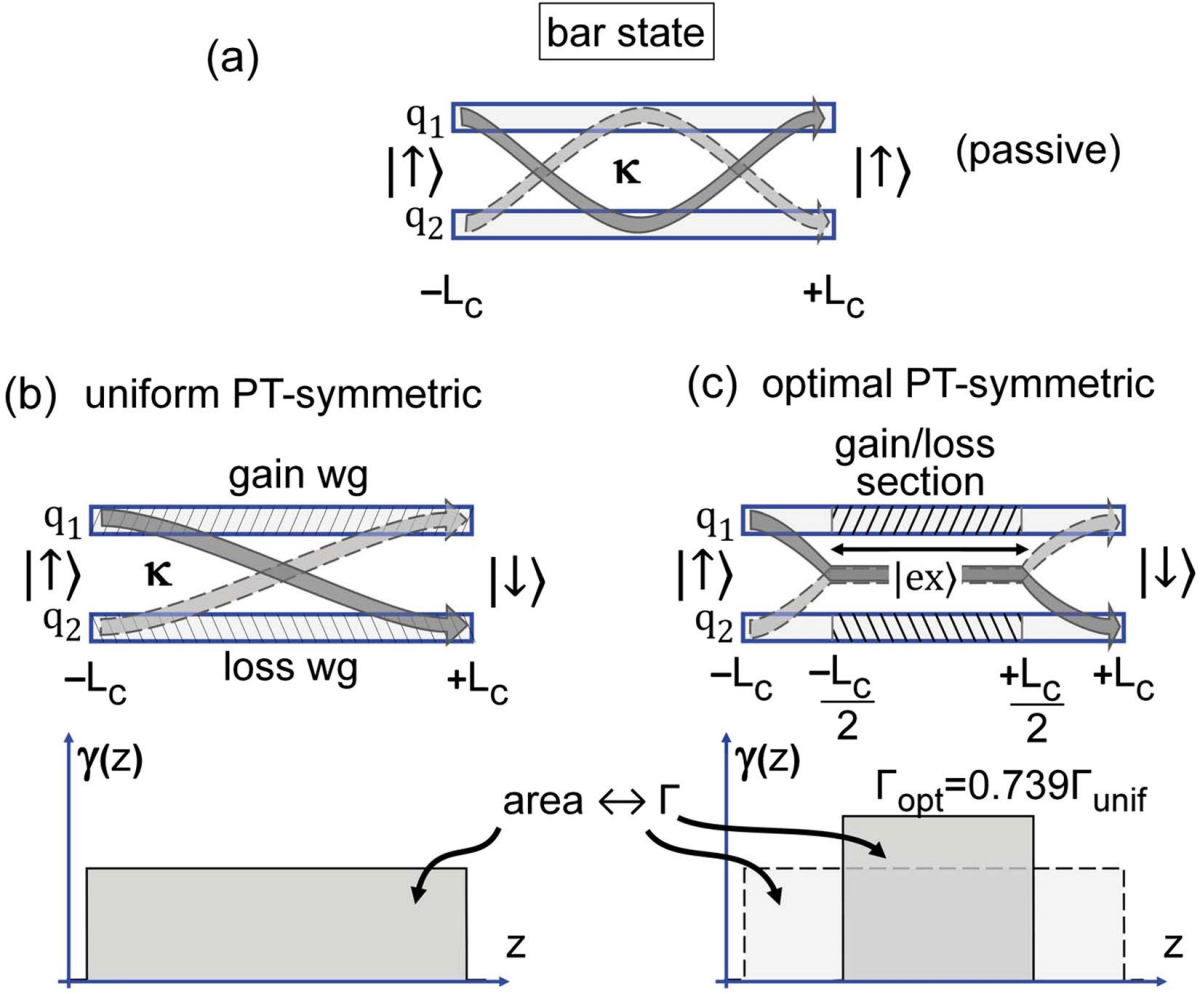
Schematic representation of (**a**) the passive coupler of the length 2*L*_*c*_ providing the bar state operation; (**b**) the uniform PT -symmetric coupler of the same length providing the cross-state operation; (**c**) the optimal coupler operating in the cross-state, which consists in combination of the two conservative segments connected by the ${\mathscr{P}}{\mathscr{T}}$-symmetric segment (dashed areas). The lower panels in (**b**) and (**c**) illustrate the energy costs in a form of the area integrals.

We are interested in the system (1) from the point of view of a switching device^18,19,26,30^. Traditionally, a four port device such as a switch is used either in the so-called bar state or in the cross state^31^. To tackle the operation of such a device in a more general language, we consider the fate and evolution of the input “binary” state $|\uparrow \rangle ={\mathrm{(1},\mathrm{0)}}^{T}$ [*T* stands for the transpose and ket and bra vectors are used for ${({q}_{1},{q}_{2})}^{T}$ and $({q}_{1}^{\ast },{q}_{2}^{\ast })$] along a non-uniform ${\mathscr{P}}{\mathscr{T}}$-symmetric system (1). Thus we consider the case when the input energy is applied to the active waveguide. We want the output of the device $z=+{L}_{c}$ to be either |↑〉 [bar state, see Fig. 1(a)] or $|\downarrow \rangle ={\mathrm{(0,}{e}^{i\phi })}^{T}$ [cross state, see Fig. 1(b,c)].

We first choose to set the bar state (|↑〉 output) as corresponding to the passive case, without gain or loss. Then we recover at the output the same signal if the coupler length obeys ${L}_{c}=\pi /2\kappa$, which binds both parameters together. Thus, this also defines that the device length in our optimization problem is 2*L*_*c*_. Under this constraint, the “cross” state (|↓〉 output) can then be achieved by introducing gain and loss in the system and we remind that our goal is to optimize Γ.

For a constant *γ*, in the unbroken ${\mathscr{P}}{\mathscr{T}}$ symmetric phase^1,2,4,18,19,30^, i.e., $\gamma  < \kappa$, we have two different real propagation constants *β*_1,2_ for the two supermodes (eigenmodes). They evolve like $\exp (i{\beta }_{\mathrm{1,2}}z)$, so that the beating length is inversely proportional to the difference ${\beta }_{2}-{\beta }_{1}=2\sqrt{{\kappa }^{2}-{\gamma }^{2}}$. As evidenced in^18^, for such an uniform ${\mathscr{P}}{\mathscr{T}}$-symmetric coupler, the amount of light amplification needed to achieve the switching operation is ${{\rm{\Gamma }}}_{unif}\approx 0.6765\times 2\pi$, corresponding to the amplification level of $\frac{10}{\mathrm{ln}\,10}{{\rm{\Gamma }}}_{unif}=18.5$ dB.

Now we look for the function $\gamma (z)$ ensuring the mapping |↑〉 → |↓〉 such that the energy cost described by Γ is minimal (we emphasize that *a priori* we do not require the ${\mathscr{P}}{\mathscr{T}}$-symmetric phase to be neither broken nor unbroken). To this end we define the Stokes components ${S}_{0}=|{q}_{1}{|}^{2}+|{q}_{2}{|}^{2}$, ${S}_{1}={q}_{1}{q}_{2}^{\ast }+{q}_{1}^{\ast }{q}_{2}$, ${S}_{2}=i({q}_{1}{q}_{2}^{\ast }-{q}_{1}^{\ast }{q}_{2})$ and ${S}_{3}=|{q}_{1}{|}^{2}-|{q}_{2}{|}^{2}$, obeying the relation ${S}_{0}^{2}={S}_{1}^{2}+{S}_{2}^{2}+{S}_{3}^{2}$. In our case gain and loss do not affect the conservation of the *S*_1_, i.e. ${\dot{S}}_{1}=0$ (what is verified by the direct differentiation). Taking into account these properties, it is convenient to introduce the normalized Stokes components ${s}_{2}(z)={S}_{2}(z)/{S}_{0}(z)$ and ${s}_{3}(z)=-{S}_{3}(z)/{S}_{0}(z)$, which obey the equations

2 $${\dot{s}}_{2}=2\kappa {s}_{3}+2\gamma (z){s}_{2}{s}_{3},\,\,{\dot{s}}_{3}=-\mathrm{2(1}-{s}_{3}^{2})\gamma (z)+2\kappa {s}_{2}\mathrm{.}$$

and satisfy the boundary conditions

3 $${s}_{2}(\pm {L}_{c})=0\quad {s}_{3}(\mp {L}_{c})=\pm 1.$$

Furthermore, since ${S}_{1}(z)\equiv 0$, we verify that for the ${\mathscr{P}}{\mathscr{T}}$-symmetric coupler (1) the relation ${s}_{2}^{2}+{s}_{3}^{2}=1$ holds. This allows us to reduce the problem to the sole equation for the “phase” $\varphi (z)\in \mathrm{[0},\pi ]$ defined by ${s}_{2}(z)=\,\sin \,[\varphi (z)]$ and ${s}_{3}(z)=\,\cos \,[\varphi (z)]$:

4 $$\dot{\varphi }=-2\gamma (z)\sin \,\varphi +2\kappa ,\quad \varphi (-{L}_{c})=\mathrm{0,}\quad \varphi ({L}_{c})=\pi \mathrm{.}$$

Eq. (4) is also known as an overdamped pendulum (where *z* plays the role of time) driven by a constant force *κ* and having a time-dependent amplitude of the periodic potential given by *γ*(*z*). This yields another physical interpretation of the formulated optimization problem: finding the dependence of the amplitude of the periodic potential shifting the overdamped pendulum’s phase by *π* during the given “time” 2*L*_*c*_ with the least possible Γ.

Let us first concentrate on the extremal *γ*(*z*) ensuring an extremum of Γ (as the second step we will prove that the profile found is indeed a minimum). To this end we notice that *γ*(*z*) can be directly expressed through $\varphi (z)$ from Eq. (4) and define an auxiliary cost one-parametric family according to the integrals ${{\rm{\Gamma }}}_{\varepsilon }={\int }_{-{L}_{c}}^{{L}_{c}}{ {\mathcal L} }_{\varepsilon }dz$, where

5 $${{\mathscr{L}}}_{\varepsilon }\equiv {(\frac{-{\dot{\varphi }}_{\varepsilon }(z)+2\kappa }{\sin [{\varphi }_{\varepsilon }(z)]})}^{1+\varepsilon }\equiv {[\gamma (z)]}^{1+\varepsilon },$$

*ε* is a positive parameter and we introduced the notation ${\varphi }_{\varepsilon }(z)$ for the extremal, i.e. for a solution of the problem (4) minimizing Γ_*ε*_. We verify that ${\rm{\Gamma }}\le {\mathrm{(2}{L}_{c})}^{\varepsilon \mathrm{/(1}+\varepsilon )}{{\rm{\Gamma }}}_{\varepsilon }^{\mathrm{1/(1}+\varepsilon )}$ for any positive *ε* [it follows from the Hölder inequality], i.e. in units such that 2*L*_*c*_ is unity, the energy cost integral is less than the cost integral Γ_*ε*_ independently on $\varepsilon$. Further, we observe that ${{\rm{\Gamma }}}_{\varepsilon }\to {\rm{\Gamma }}$ in the limit $\varepsilon \to 0$. This allows us to concentrate on this last limit. Then the equation for the extremal Γ_*ε*_ is obtained as a solution of the Euler-Lagrange equation $(d/dz)(\partial {L}_{\varepsilon }/\partial \dot{\varphi })=\partial {L}_{\varepsilon }/\partial \varphi$.

The usefulness of the above generalization (to nonzero *ε*) stems from the fact that at $\varepsilon =0$ the Euler-Lagrange equation takes the form $\cos \,{\varphi }_{0}/{\sin }^{2}{\varphi }_{0}=0$ and can be satisfied only by the constant ${\varphi }_{{\rm{ex}}}\equiv \pi \mathrm{/2}$ and thus *does not* have the extremal ${\varphi }_{0}(z)$ with the fixed boundary points of (4). Therefore, to find ${\varphi }_{0}(z)$ [and consequently $\gamma (z)$] we turn to the minimization problem for Γ_*ε*_.

Before proceeding, we notice that ${\varphi }_{{\rm{ex}}}\equiv \pi /2$ on the one hand corresponds to the state $|{\rm{e}}x\rangle ={\mathrm{(1},-i)}^{T}$ possibly within a phase factor, and is *only* possible for the *exceptional point*, i.e. when $\gamma (z)\equiv \kappa$ on some interval of *z*.

For $\varepsilon  > 0$, the extremal ${\varphi }_{\varepsilon }$ can be found directly from the “Hamiltonian” [computed as ${ {\mathcal H} }_{\varepsilon }=(\partial { {\mathcal L} }_{\varepsilon }/\partial \dot{\varphi })$ $\dot{\varphi }-{ {\mathcal L} }_{\varepsilon }$]:

6 $${ {\mathcal H} }_{\varepsilon }={({\dot{\varphi }}_{\varepsilon }+2\kappa )}^{\varepsilon }(\varepsilon {\dot{\varphi }}_{\varepsilon }-2\kappa )/{\sin }^{1+\varepsilon }{\varphi }_{\varepsilon }$$

which is *z*-independent. Since $\sin ({\varphi }_{\varepsilon })$ cannot identically vanish, ${ {\mathcal H} }_{\varepsilon }$ is a finite constant. However, this sine has to vanish at boundaries, and we can exploit this to deduce the form of the extremal solution. Considering the limit $z\to \pm {L}_{c}$ and using the fixed point boundary conditions for *ϕ* [see (4)] we obtain that the only way for (6) to be finite in spite of the vanishing denominator is that ${\dot{\varphi }}_{\varepsilon }(\pm {L}_{c})=-2\kappa$ and more precisely that ${({\dot{\varphi }}_{\varepsilon }+2\kappa )}^{\varepsilon }\sim {\sin }^{1+\varepsilon }{\varphi }_{\varepsilon }$. This leads to an important conclusion: for the gain-and-loss minimizing the gain and loss must be zero at the input and output of the coupler, i.e. $\gamma (\pm {L}_{c})=0$ for any $\varepsilon  > 0$. Furthermore, supposing near the ends of the coupler $\gamma \sim {(z\mp {L}_{c})}^{\nu }$ where $\nu  > 0$ [i.e. excluding distributions $\gamma (z)$ approaching zero beyond all orders, as experimentally not feasible] we obtain the asymptotic ${H}_{\varepsilon }\sim {(z\mp {L}_{c})}^{\varepsilon \nu -1}$ at $z\to \pm {L}_{c}$, what is possible only at $\nu =1/\varepsilon$. Thus the solution $\gamma (z)$ of the optimization problem must decay faster than any positive power of $(z\mp {L}_{c})$.

Thus, taking into account the ${\mathscr{P}}{\mathscr{T}}$-symmetry of the problem, the gain-and-loss distribution we are interested in is symmetric with respect to $z=0$ and at *ε* = 0 obeys the property $\gamma (z)\equiv 0$ at $z\in [-{L}_{c},-{L}_{c}+\ell ]\cup$ $[{L}_{c}-\ell ,{L}_{c}]$ (below in Methods, we present a proof that to fulfill the condition of binary states at the input and output, the gain and loss distributions must be symmetric). In other words the device we are interested in must start and end up with conservative elements, whose length $\ell$ is to be found. Another important consequence of the assumption about conservative propagation close to input and output is that ${ {\mathcal H} }_{\varepsilon }=0$, which is in apparent contradiction to the non-conservative propagation along the interval $z\in [-\ell ,\ell ]$. This contradiction is resolved if we allow $\gamma (z)$ to be a discontinuous function. Indeed, in this case we have that ${\rm{\Gamma }}={\int }_{-\ell }^{\ell }\gamma (z)dz$ which must be minimized now in the interval $z\in [-\ell ,\ell ]$, with the fixed boundary conditions that can be chosen different form those defined in (4). This last problem, was already solved above when considering $\varepsilon =0$: the extremum is achieved by the constant $\gamma =\kappa$ corresponding to the exceptional point. To this end, however we have to ensure that the conservative parts at the input and output of the coupler perform the transformations $|\uparrow \rangle \to |\mathrm{ex}\rangle$ and $|{\rm{ex}}\rangle \to |\downarrow \rangle$. To resolve this last issue it is enough to choose $\ell ={L}_{c}/2$, which defines the length of the conservative parts, and sets that of the ${\mathscr{P}}{\mathscr{T}}$ symmetric part as *L*_*c*_.

Finally, the gain-and-loss distribution reads

7 $$\gamma (z)\equiv \{\begin{array}{cc}0, & \,{\rm{a}}{\rm{t}}\,z\in [-{L}_{c},-{L}_{c}/2]\cup [{L}_{c}/2,{L}_{c}]\,\\ \kappa , & \,{\rm{a}}{\rm{t}}\,z\in [-{L}_{c}/2,{L}_{c}/2]\,\end{array}$$

what corresponds to ${\rm{\Gamma }}={{\rm{\Gamma }}}_{{\rm{opt}}}=\pi /2$. This distribution is illustrated in Fig. 1(c).

To complete the solution of the minimization problem we only need to show that the extremal solution does correspond to the minimum of Γ. Since, the conservative parts correspond to zero costs, to prove that *γ* achieves its minimum on (7) we first consider small deviations of the length and the strength of the ${\mathscr{P}}{\mathscr{T}}$-symmetric segment. These deviations are not arbitrary, however, but must respect the continuity of the field $\varphi (z)$. We drop tedious but straightforward algebra and just indicate the result. Characterizing a small deviation of *γ* from the exceptional point value *κ* by *δ*, which is defined through the relation $\gamma =\kappa \sqrt{1-\delta }$, we obtain $\ell /{L}_{c}=1/2+\delta$ that gives ${{\rm{\Gamma }}/{\rm{\Gamma }}}_{{\rm{opt}}}=1+{\pi }^{2}{\delta }^{2}/24+{\mathscr{O}}({\delta }^{4})$, i.e. the extremal solution found in (7) indeed provides the minimal value for the energy costs.

## Discussion

What has the optimization brought us? The gain-loss distribution [Fig. 1(c)] found through optimization procedure corresponds the lowest amount of cumulated gain required for switching operation. We get the switching operation for only 0.5 × 2*π*. The remarkable point is that in contrast to the case of uniform ${\mathscr{P}}{\mathscr{T}}$-symmetric coupler, the switching operation is obtained for ≈5 dB lower amplification level (from 18.5 to 13.6 dB), thus, for practitioners, a diminished optical amplification by a factor more than three.

**Figure 2:**
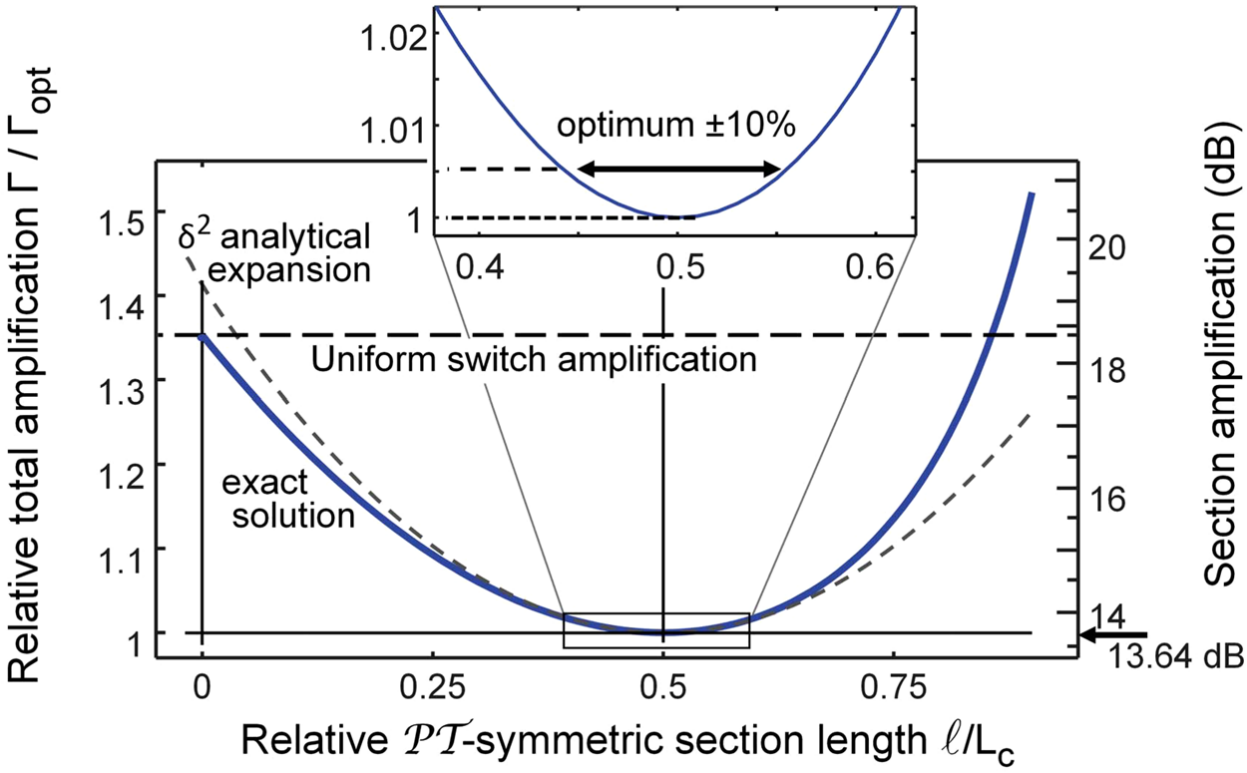
The relative total amplification ${{\rm{\Gamma }}/{\rm{\Gamma }}}_{{\rm{opt}}}$ *vs*. normalized length of the ${\mathscr{P}}{\mathscr{T}}$-symmetric segment. The inset shows the parabolic behavior near the optimal value ${{\rm{\Gamma }}}_{{\rm{opt}}}$.

The Taylor expansion used for the analytical proof of the minimum also hints at the tolerance, which is the practically relevant characteristic, for an actual device design. We address the issue numerically, by calculating with conventional coupled mode theory the amplification required for switching for various values of the parameter $\ell$ defining the lengths of the passive and ${\mathscr{P}}{\mathscr{T}}$ symmetric section of the switch. The results displayed in Fig. 2 show that there is a broad minimum around the optimal value $\ell ={L}_{c}/2$. The numerical data reproduce the quadratic dependence predicted by theory with the coefficient ${\pi }^{2}\mathrm{/24}\approx 0.41123$. In terms of parameter tolerance, we find that we increase the figure of merit Γ by only 0.5% (0.065 dB) when we change $\ell$ by 10%. Our switch device should thus be very tolerant, in terms of optimality, with respect to most fabrication imperfections commonly encountered in integrated optics.

Mathematical estimates of field perturbations due to imperfections of the ${\mathscr{P}}{\mathscr{T}}$-symmetric segment show that they do not exceed values of order of ~26 *μ/κ* (see Methods below), with *μ* defined as the largest deviation of parameters from the exceptional point values: $\mu ={{\rm{\max }}}_{z}\{|\kappa (z)-\kappa |,|\gamma (z)-\gamma |\}$ for arbitrarily shaped deviations of $\kappa (z)$ and $\gamma (z)$ from their average values *κ* and *γ*.

**Figure 3:**
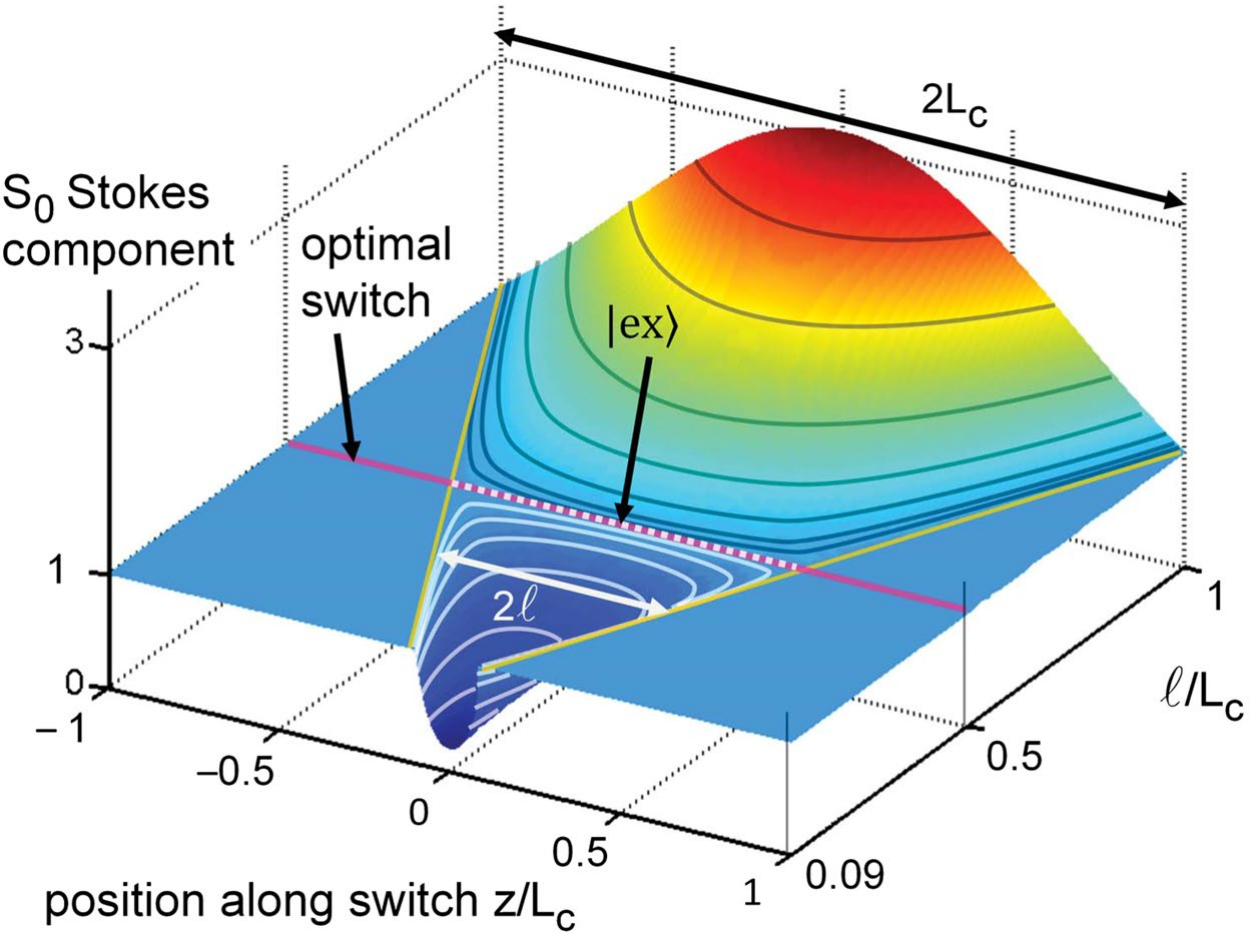
*S*_0_ Stokes component as a function of position $z/{L}_{c}$ and of relative ${\mathscr{P}}{\mathscr{T}}$-symmetric section length $\mathrm{(2}\ell \mathrm{)/(2}{L}_{c})$, whose limits are thus the oblique lines starting at the rear corners. The line at $\ell /{L}_{c}=0.5$ is the case of optimal gain-loss profile, conserving *S*_0_. The exceptional point state $|{\rm{ex}}\rangle$ ensures transit in the $-\ell  < z < \ell$ region (added white dots).

It is insightful to visualize the field distribution inside the switch for situations around the optimal one. We display in Fig. 3 a colormap of *S*_0_ as a function of normalized coordinate $z/{L}_{c}$ and fractional length of ${\mathscr{P}}{\mathscr{T}}$-symmetric segment $\mathrm{(2}\ell \mathrm{)/(2}{L}_{c})$ in the cross state (when parameters are such that the output is $|\downarrow \rangle$). In general, the energy is not conserved in the central segment. A large swing of the energy appears notably in the case $\ell ={L}_{c}$, corresponding to the case of the uniform ${\mathscr{P}}{\mathscr{T}}$-symmetric coupler, with *S*_0_ gently peaking above 3 at $z=0$, due to one component being boosted during the transit. The optimal trajectory, remarkably, corresponds to the energy conserving case ${S}_{0}=1$: we see that the overshoot of *S*_0_ vanishes in the optimal state. If we reduce $\ell$ further, a converse trend occurs on *S*_0_, as the waveguide amplitude boosted in the central segment is now the other one compared to the case $\ell  < {L}_{c}$. So we have the coincidence of optimality, energy conservation, and operation of the coupler’s central part in the exceptional point regime.

Having looked into the device “inner” behavior, let us come back to the global view of our optimization problem. One might first wonder why we chose the optimization of the gain-and-loss profile, rather than optimization through the coupling constant. We checked that it is achievable, but the optimum leads to infinities for *κ* (Dirac distributions) not tractable in a feasible device.

Next, let us underline the remarkable properties of the obtained optimization. Firstly, it is achieved by *combination of conservative and non-Hermitian evolution* of the field. Secondly, in both conservative and ${\mathscr{P}}{\mathscr{T}}$-symmetric parts, propagation occurs with *conserved energy*, i.e. with a constant Stokes component *S*_0_. Thus the optimal coupler, requiring the minimal energy costs contains the conservative parts performing transformation between the input (output) binary states and the internal state, which is conserved at the exceptional point, this phase “freezing” being ensured by the ${\mathscr{P}}{\mathscr{T}}$-symmetric section. Thirdly, the optimal non-Hermitian evolution has to occur *right at the exceptional point of the device* which preserves the state along the evolution and thus ensures the required freezing of the phase difference needed for the cross state in the central part. Here, there is a counter-intuitive aspect as on the one hand, the exceptional point is a point of maximal eigenvalues sensitivity to system parameters, but on the other hand, in our combination of conservative and ${\mathscr{P}}{\mathscr{T}}$-symmetric structure, there is a large design tolerance as discussed above. From the argument of phase freezing, we can also infer that the obtained operation principle holds for longer devices $L > {L}_{c}$, using “freezing sections” of adequate length to ensure both bar and cross states, but defining the figure of merit is less obvious.

To conclude, the optimization problem as considered here goes beyond the standard class of brachistochrone-like problems (quantum or classical). Our considerations are not restricted to ${\mathscr{P}}{\mathscr{T}}$-symmetric systems that occupy an intermediate position between Hermitian and non-Hermitian systems in the quantum case^3^, and between Hamiltonian and dissipative systems in the classical case^4^. This problem is of particular relevance for really open systems, like those involving scattering and decay processes^15^ or the spin flipping, where instead of time being the figure-of-merit for the brachistochrone problem, one would consider the energy supplied to the system. The connection to preparation of controlled entangled state for quantum information (see e.g^32^.) is yet another possible avenue whereby the combination with an optimization approach like ours, which protects information in the steady state of the exceptional point, could offer several advantages for efficient quantum control when operating at large rates. Finally, even in the classical statement, the considered optimization problem can be applied not only to optical systems as the switch exemplified above: we have already shown that it addresses the overdamped driven pendulum.

A common thread to these problems is the obtainment of energy minimization in controllable systems, an important feature in our era where the issue of taming energy costs pervades across the whole spectrum of information technologies. We thus believe that the combination of non-Hermitian Hamiltonians with conservative ones will address an increasingly large class of relevant physical problems as well as their practical applications.

## Methods

Above we argued qualitatively that weak imperfections of the coupler, which affect the exact matching of gain and losses and result in a shift of the system from the exceptional point in the parameter space, do not significantly affect the energy cost integral. We also addressed the gain and losses distributed symmetrically with respect to the input and output of the coupler. In this Section we present mathematical proofs of both claims.

### Justification of the symmetrical distribution of the gain and losses

First of all, we justify mathematically that the required input and output signals imply a symmetric distribution of the gain and loss profiles. To this end we rewrite system (1) in a form

8 $$\frac{d\chi }{dz}=(\begin{array}{cc}i\kappa  & \gamma (z)\\ \gamma (z) & -i\kappa \end{array})\chi ,\quad \quad \chi (z)=(\begin{array}{c}{\chi }_{1}(z)\\ {\chi }_{2}(z)\end{array})\equiv (\begin{array}{c}{q}_{1}-{q}_{2}\\ {q}_{1}+{q}_{2}\end{array})$$

When *κ* is considered as a formal spectral parameter the system (8) becomes the well-known as Zakharov-Shabat spectral problem^33^. The column-vector *χ* must satisfy the boundary conditions

9 $$\chi (-{L}_{c})=(\begin{array}{c}{e}^{-i\phi }\\ {e}^{-i\phi }\end{array}),\quad \quad \chi ({L}_{c})=(\begin{array}{c}-{e}^{i\phi }\\ {e}^{i\phi }\end{array}),$$

where $2\phi$ is a real constant, which is a constant phase shift between the output and input signals. Obviously, these conditions correspond to the input |↑〉 and |↓〉 binary states at the input and output.

Let us now extend our coupler beyond the interval $[-{L}_{c},{L}_{c}]$ by pure conservative arms (alternatively one can consider the limit ${L}_{c}\to \infty$ with $\gamma (z)\to 0$ at $|z|\to {\rm{\infty }}$). Since $\gamma (z)\equiv 0$ at $|z| > {L}_{c}$, we can consider the Jost solutions defined by the asymptotics

10 $${{\rm{\Phi }}}_{1}\to (\begin{array}{c}{e}^{i\kappa z}\\ 0\end{array}),\quad {{\rm{\Phi }}}_{2}\to (\begin{array}{c}0\\ {e}^{-i\kappa z}\end{array}),\quad z\to -{\rm{\infty }};\quad \quad {{\rm{\Psi }}}_{1}\to (\begin{array}{c}{e}^{i\kappa z}\\ 0\end{array}),\quad {{\rm{\Psi }}}_{2}\to (\begin{array}{c}0\\ {e}^{-i\kappa z}\end{array}),\quad z\to +{\rm{\infty }}$$

The Jost solutions are connected by the the transfer matrix

11 $$T(\phi ,\kappa )=(\begin{array}{cc}{a}^{\ast }(\phi ,\kappa ) & {b}^{\ast }(\phi ,\kappa )\\ b(\phi ,\kappa ) & a(\phi ,\kappa )\end{array})$$

through the formula ${{\rm{\Phi }}}_{j}={T}_{j1}{{\rm{\Psi }}}_{1}+{T}_{j2}{{\rm{\Psi }}}_{2}$ where ${T}_{ji}$ with $i,j=1,2$ are the entries of *T*. Since a pair of the Jost solutions represents a complete basis, there exist coefficients $\alpha$, $\beta$, $\sigma$ and $\delta$, such that $\chi =\alpha {{\rm{\Phi }}}_{1}+\beta {{\rm{\Phi }}}_{2}=\sigma {{\rm{\Psi }}}_{1}+\delta {{\rm{\Psi }}}_{2}$. Considering this last expression at $z=\pm {L}_{c}$ one readily finds the relations

12 $$\sigma ={\alpha }^{\ast }={e}^{i(\phi +\kappa {L}_{c})},\quad \quad \delta =-{\beta }^{\ast }={e}^{i(\phi -\kappa {L}_{c})}$$

Furthermore, since $|a{|}^{2}-|b{|}^{2}=1$, expressing ${{\rm{\Psi }}}_{j}$ through ${{\rm{\Phi }}}_{k}$ in the formula for $\chi$, one finds that the compatibility of all the relations require

13 $${({\sigma }^{\ast })}^{2}{b}^{\ast }=-{\sigma }^{2}b\quad \quad \,{\rm{and}}\,\quad \quad {({\delta }^{\ast })}^{2}{b}^{\ast }=-{\delta }^{2}b$$

These last two conditions impose the constraint on the phase mismatch, requiring $\phi ={\phi }_{n}=\pi n/4$. Finally we make use of the imposed condition $\kappa {L}_{c}=\pi$, as well as the symmetry relation of the coefficient $b({\phi }_{n},\kappa )$ with respect to the change of the sign of *κ* ^34^, and obtain

14 $${b}^{\ast }({\phi }_{n},\kappa )={(-\mathrm{1)}}^{n}b({\phi }_{n},\kappa )=b({\phi }_{n},-\kappa \mathrm{).}$$

From this formula and using Theorems 5 and 6 from^34^, we conclude that the required switch between the binary states with the phase difference $2{\phi }_{2p+1}=(p+1/\mathrm{2)}\pi$ the gain-loss coefficient $\gamma (z)$ must be even, $\gamma (z)=\gamma (-z)$, while for the phase difference $2{\phi }_{2p}=p\pi$ the gain-loss profile must be odd, $\gamma (z)=-\gamma (-z)$.

### On limited impact of imperfectnesses

To estimate the general effect of weak imperfectnesses we observe that the ${\mathscr{P}}{\mathscr{T}}$-symmetric part of the coupler, where the matching problem can occur, occupies a symmetric segment of length $2{L}_{c}$ (as proven above). Next, we make use of the coupler equations (1) in the ${\mathscr{P}}{\mathscr{T}}$-symmetric section when operating at the exceptional point ($\kappa =\gamma$). They can be rewritten in the matrix form

15 $$i\dot{{\bf{q}}}={H}_{0}{\bf{q}},\quad {\bf{q}}=(\begin{array}{c}{q}_{1}\\ {q}_{2}\end{array}),\quad {H}_{0}=\kappa (\begin{array}{cc}i & 1\\ 1 & -i\end{array}).$$

We also use the fact that the signal impinges at the ${\mathscr{P}}{\mathscr{T}}$-symmetric part of the coupler in the state

16 $${{\bf{q}}}_{0}={\mathrm{(1,}-i)}^{T}$$

(possibly with a pure phase factor). Notice that for the sake of convenience hereafter we shifted the origin: we consider the coupled waveguides located on the interval $z\in \mathrm{[0},2{L}_{c}]$, instead of $[-{L}_{c},{L}_{c}]$ used above.

Now we assume that the system is operating not in the exact exceptional point described by *H*_0_, but that deviations of the parameters, i.e. of the coupling, gain, and loss, are relatively small. We describe these deviations by a matrix $\mu {H}_{1}(z)$, whose entries ${h}_{ij}$ may depend on *z*, but are bounded:

17 $$|{h}_{ij}(z)| < \mathrm{1,}\quad i,j=1,2.$$

The small parameter $\mu \ll 1$ characterizes the strength of the imperfections. Thus, the propagation in the imperfect coupler, instead of (15) must now be found by solving the system

18 $$i\dot{{\bf{q}}}=H{\bf{q}},\quad \quad H={H}_{0}+\mu {H}_{1}$$

which is fed by the initial conditions ${\bf{q}}(z=\mathrm{0)}={{\bf{q}}}_{0}$.

Now we look for a solution of (18) in the form of the expansion ${\bf{q}}={{\bf{q}}}_{0}+\mu {{\bf{q}}}_{1}+\cdots$ In the leading order of *μ* we have ${{\bf{q}}}_{0}=0$ and ${H}_{0}{{\bf{q}}}_{0}=0$. In the first order we compute

19 $$i{\dot{{\bf{q}}}}_{1}={H}_{0}{{\bf{q}}}_{1}+{H}_{1}{{\bf{q}}}_{0},\quad {{\bf{q}}}_{1}(z=\mathrm{0)}=0.$$

This equation is readily solved. Using the notations ${f}_{1}(z)={h}_{11}-i{h}_{12}$, and ${f}_{2}(z)={h}_{21}-i{h}_{22}\,,$ as well as ${{\bf{q}}}_{1}={({q}_{11},{q}_{21})}^{T}$, we write down the solution in the form

20 $$\begin{array}{ccc}{q}_{11} & = & i\kappa {\int }_{0}^{z}dy{\int }_{0}^{y}dx\,[i{f}_{2}(x)-{f}_{1}(x)]\,-i\,{\int }_{0}^{z}{f}_{1}(x)dx,\\ {q}_{21} & = & \kappa {\int }_{0}^{z}dy{\int }_{0}^{y}dx\,[i{f}_{2}(x)-{f}_{1}(x)]-i{\int }_{0}^{z}{f}_{2}(x)dx\end{array}$$

Taking into account (17), we deduce that $|\,{f}_{\mathrm{1,2}}|\le 2$. Hence the last formulas yield the estimates: $|{q}_{11}|\,,|{q}_{21}$ $|\le 2\kappa {z}^{2}+2z\mathrm{.}$

Thus, independently of the particular type of *z*-dependent perturbation of the exceptional point matrix *H*_0_, the maximal possible relative downstream perturbation of the solution **q**_0_ (or $|{\rm{ex}}\rangle$ state introduced in the main text) at the input of the ${\mathscr{P}}{\mathscr{T}}$-symmetric segment, does not exceed the value

21 $$4\mu {L}_{c}\mathrm{(2}{L}_{c}\kappa +\mathrm{1)}=2({\pi }^{2}+\pi )\frac{\mu }{\kappa }\approx 26.022\frac{\mu }{\kappa }$$

at the output (here we use that $2{L}_{c}=\pi /\kappa$).
